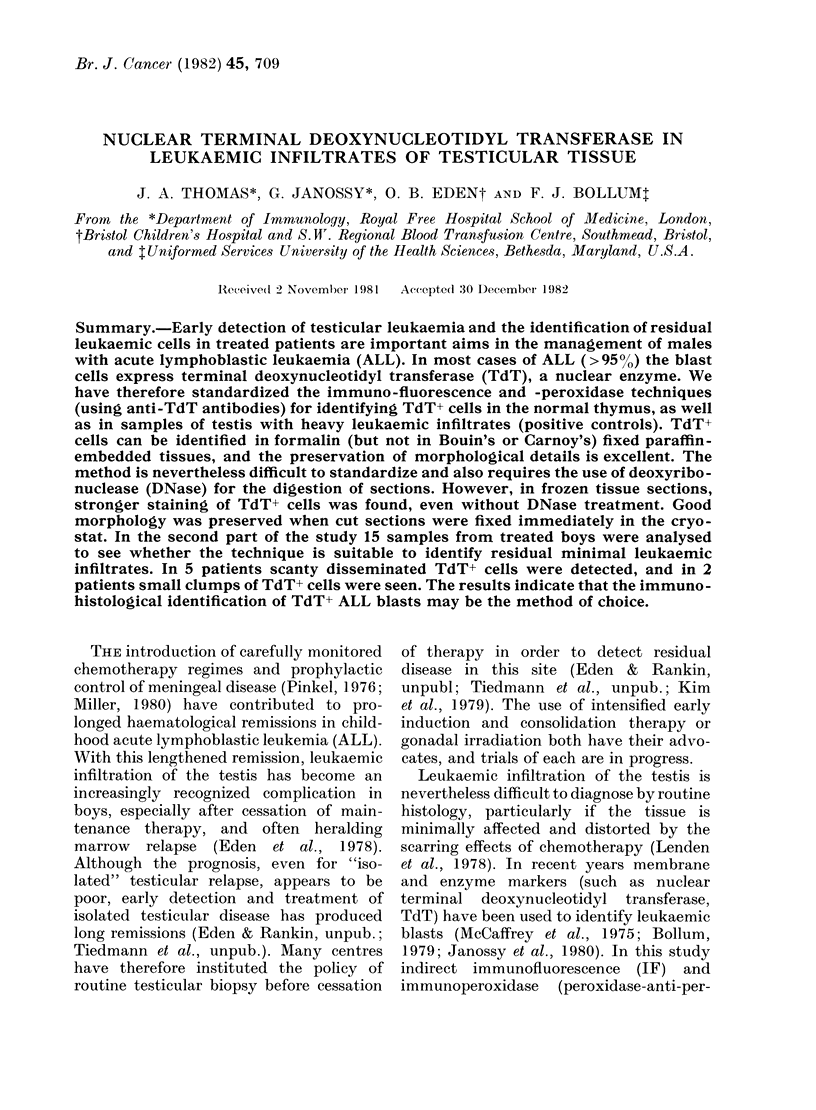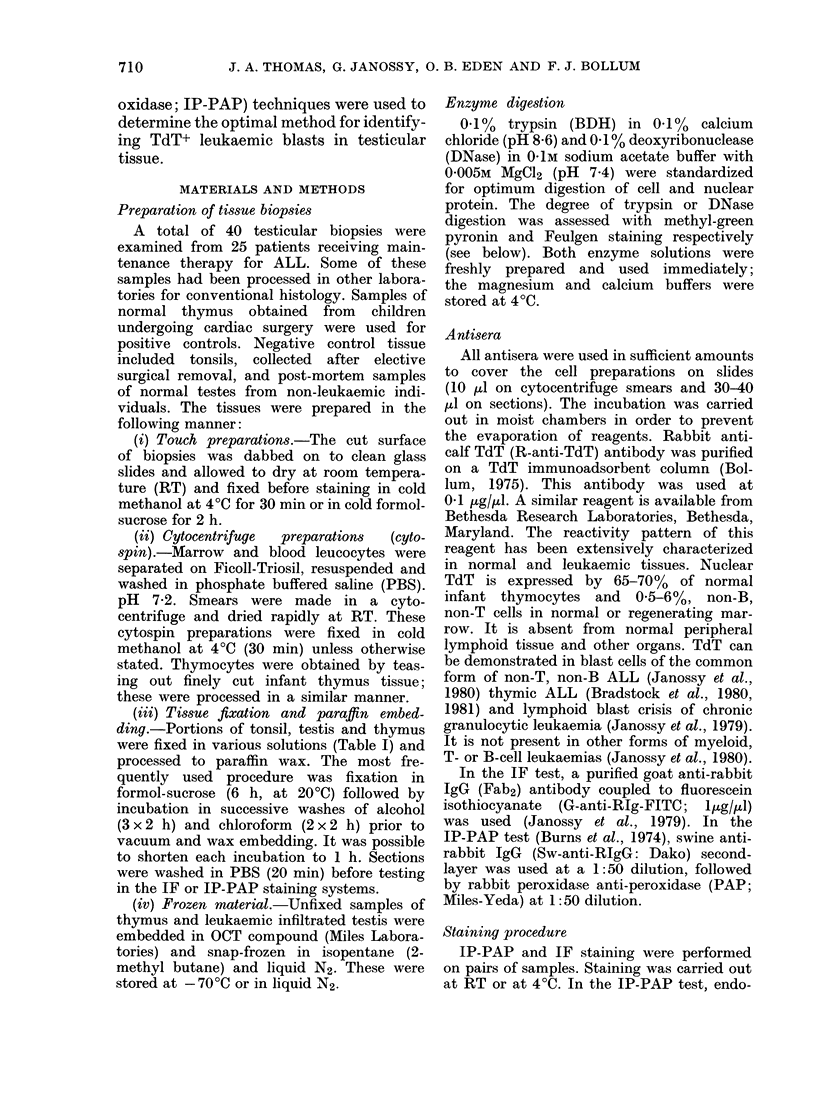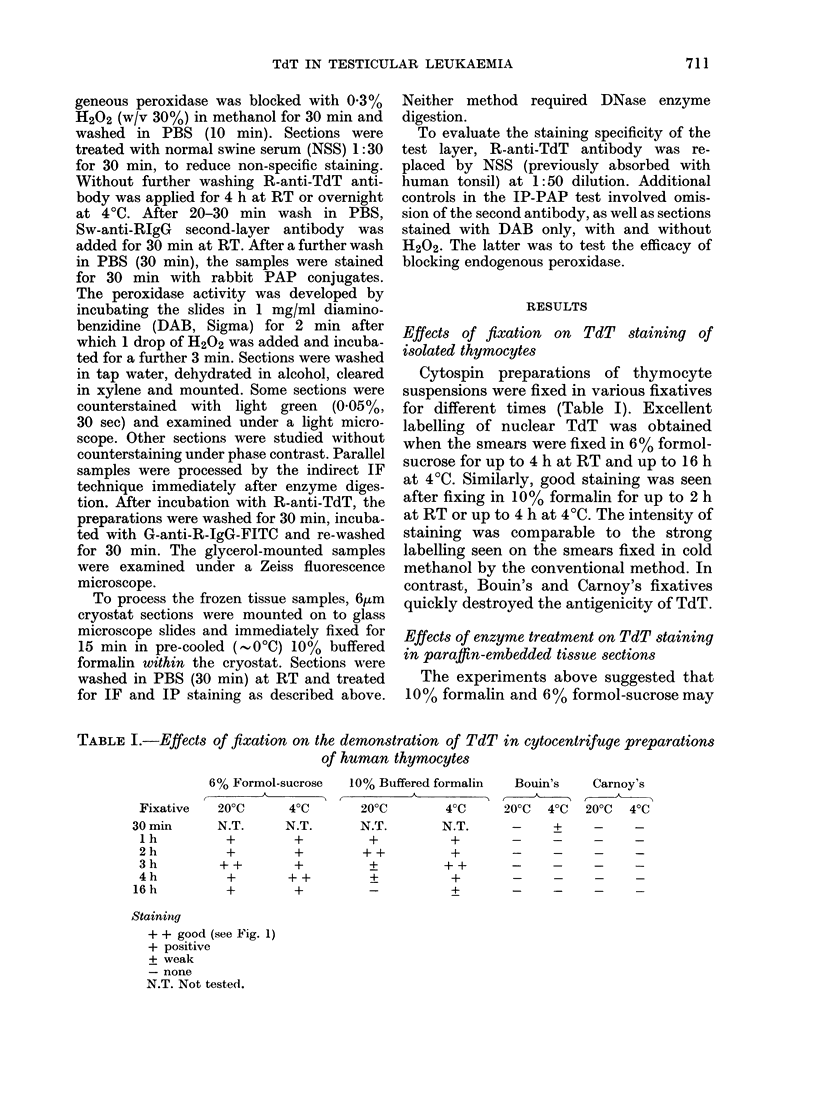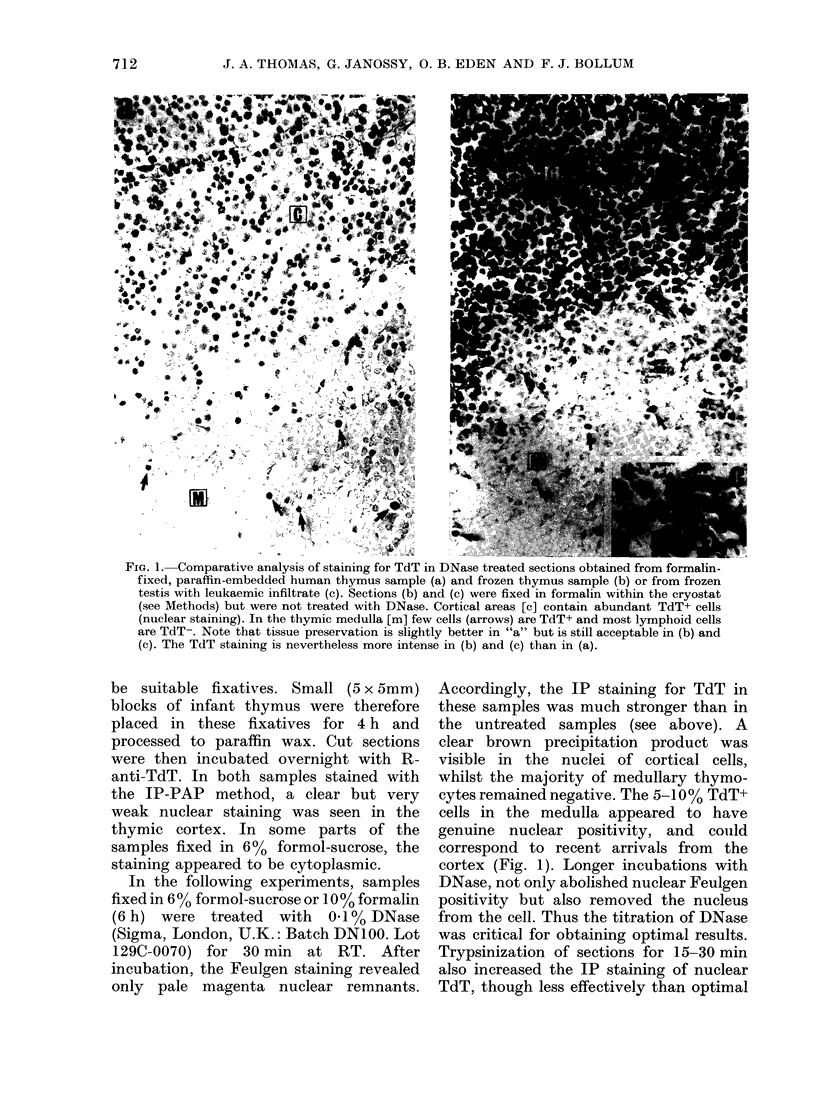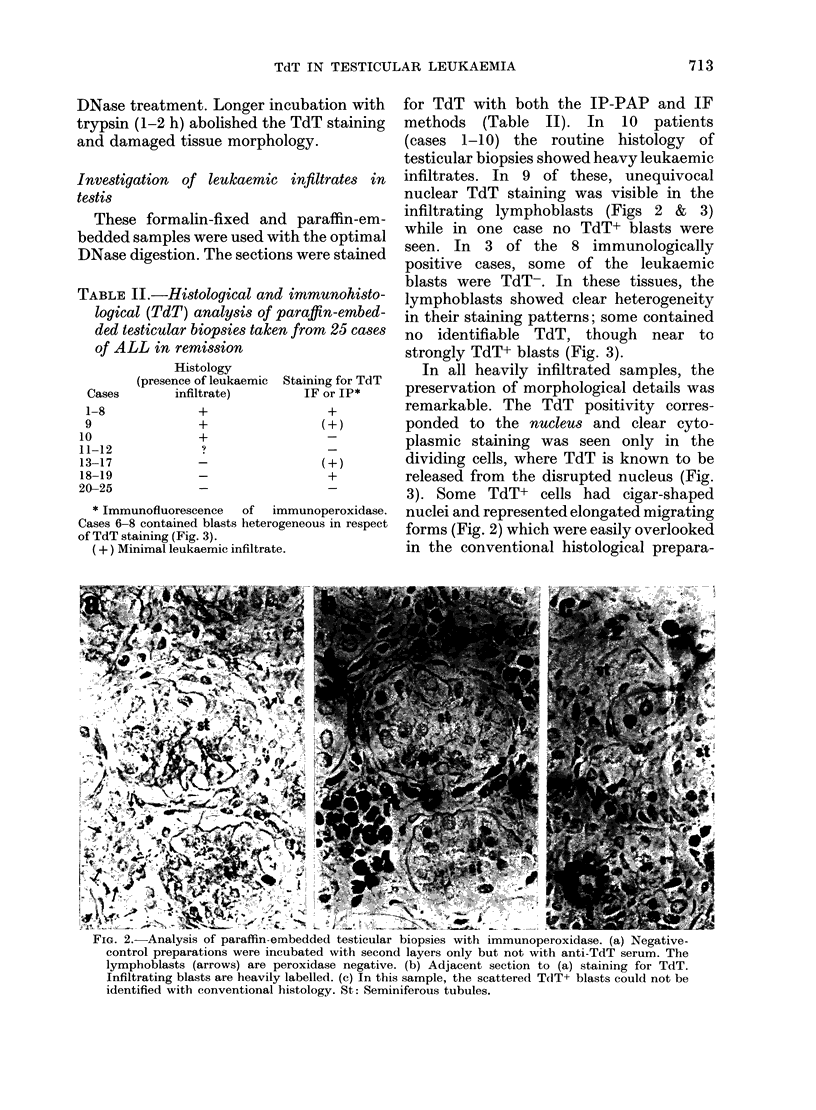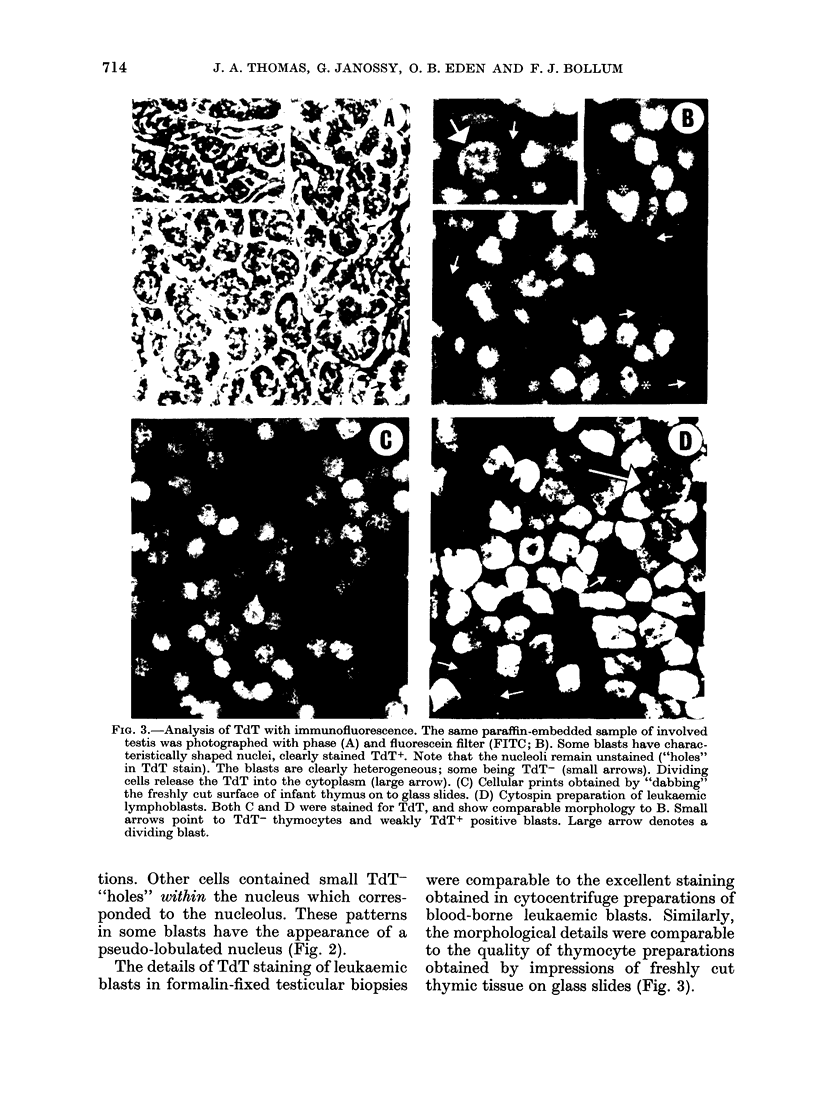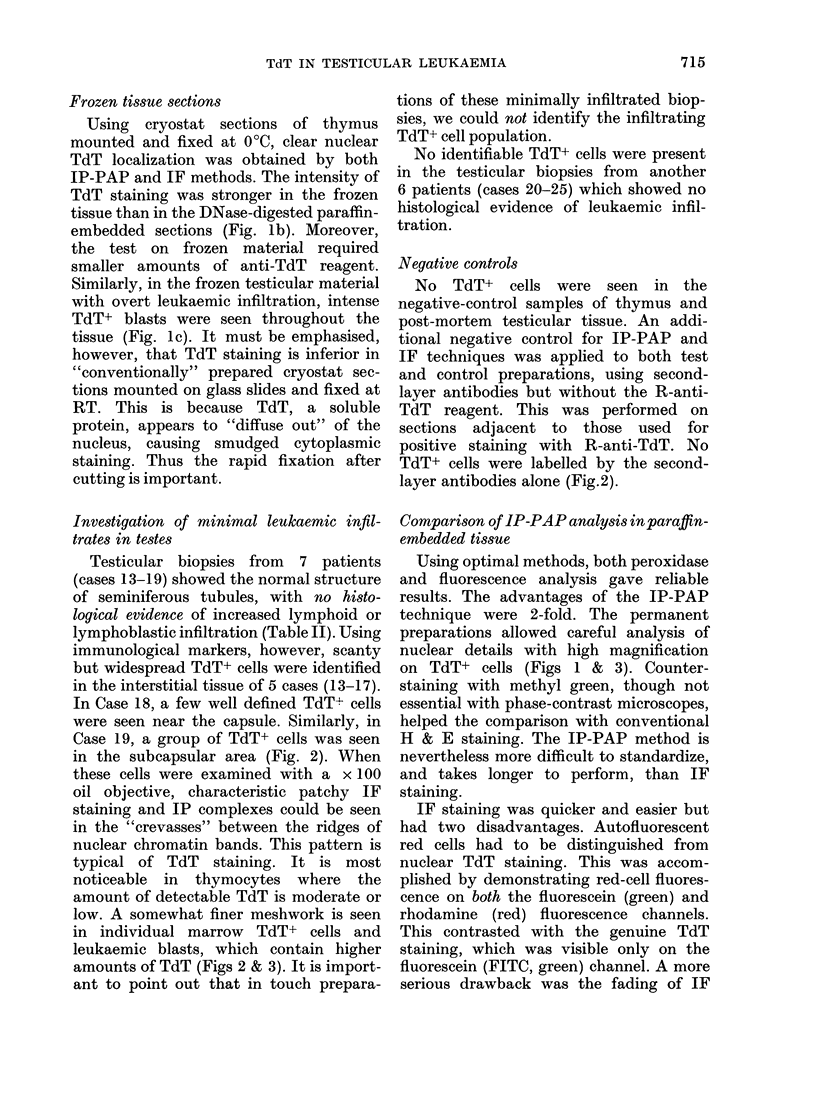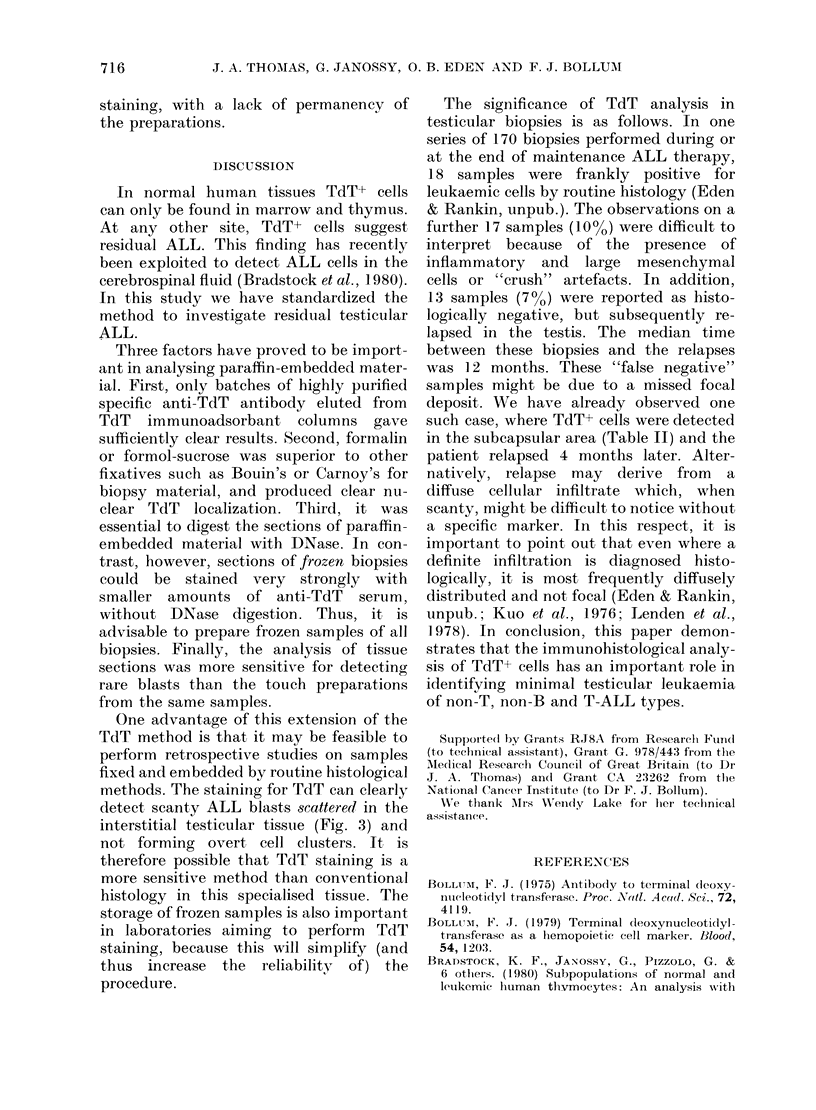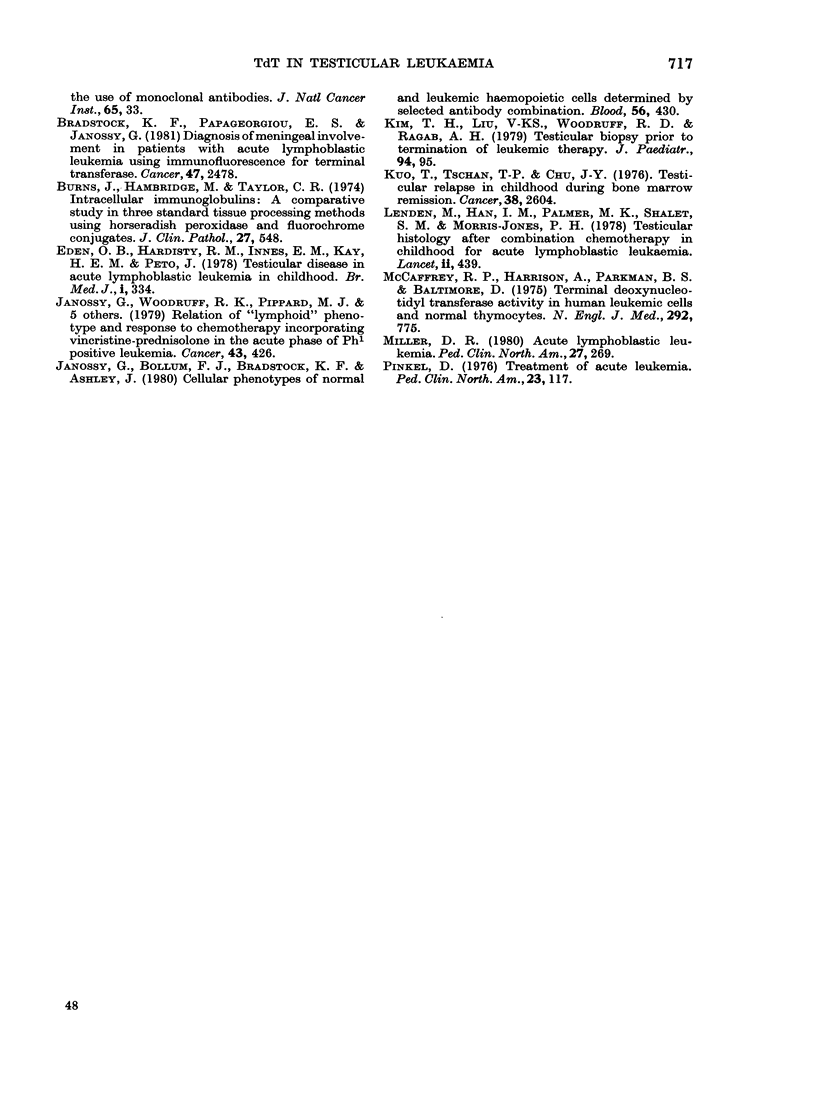# Nuclear terminal deoxynucleotidyl transferase in leukaemic infiltrates of testicular tissue.

**DOI:** 10.1038/bjc.1982.112

**Published:** 1982-05

**Authors:** J. A. Thomas, G. Janossy, O. B. Eden, F. J. Bollum

## Abstract

**Images:**


*Br. J. Cancer* (1982) **45**, 709

### NUCLEAR TERMINAL DEOXYNUCLEOTIDYL TRANSFERASE IN LEUKAEMIC INFILTRATES OF TESTICULAR TISSUE

J. A. THOMAS*, G. JANOSSY*, O. B. EDEN† AND F. J. BOLLUM‡

*From the *Department of Immunology, Royal Free Hospital School of Medicine, London, †Bristol Children's Hospital and S.W. Regional Blood Transfusion Centre, Southmead, Bristol, and ‡Uniformed Services University of the Health Sciences, Bethesda, Maryland, U.S.A.*

Received 2 November 1981 Accepted 30 December 1982

**Summary.—Early detection of testicular leukaemia and the identification of residual leukaemic cells in treated patients are important aims in the management of males with acute lymphoblastic leukaemia (ALL). In most cases of ALL (>95%) the blast cells express terminal deoxynucleotidyl transferase (TdT), a nuclear enzyme. We have therefore standardized the immuno-fluorescence and -peroxidase techniques (using anti-TdT antibodies) for identifying TdT$^+$ cells in the normal thymus, as well as in samples of testis with heavy leukaemic infiltrates (positive controls). TdT$^+$ cells can be identified in formalin (but not in Bouin's or Carnoy's) fixed paraffin-embedded tissues, and the preservation of morphological details is excellent. The method is nevertheless difficult to standardize and also requires the use of deoxyribonuclease (DNase) for the digestion of sections. However, in frozen tissue sections, stronger staining of TdT$^+$ cells was found, even without DNase treatment. Good morphology was preserved when cut sections were fixed immediately in the cryostat. In the second part of the study 15 samples from treated boys were analysed to see whether the technique is suitable to identify residual minimal leukaemic infiltrates. In 5 patients scanty disseminated TdT$^+$ cells were detected, and in 2 patients small clumps of TdT$^+$ cells were seen. The results indicate that the immunohistological identification of TdT$^+$ ALL blasts may be the method of choice.**

THE introduction of carefully monitored chemotherapy regimes and prophylactic control of meningeal disease (Pinkel, 1976; Miller, 1980) have contributed to prolonged haematological remissions in childhood acute lymphoblastic leukemia (ALL). With this lengthened remission, leukaemic infiltration of the testis has become an increasingly recognized complication in boys, especially after cessation of maintenance therapy, and often heralding marrow relapse (Eden *et al.*, 1978). Although the prognosis, even for "isolated" testicular relapse, appears to be poor, early detection and treatment of isolated testicular disease has produced long remissions (Eden & Rankin, unpub.; Tiedmann *et al.*, unpub.). Many centres have therefore instituted the policy of routine testicular biopsy before cessation of therapy in order to detect residual disease in this site (Eden & Rankin, unpubl; Tiedmann *et al.*, unpub.; Kim *et al.*, 1979). The use of intensified early induction and consolidation therapy or gonadal irradiation both have their advocates, and trials of each are in progress.

Leukaemic infiltration of the testis is nevertheless difficult to diagnose by routine histology, particularly if the tissue is minimally affected and distorted by the scarring effects of chemotherapy (Lenden *et al.*, 1978). In recent years membrane and enzyme markers (such as nuclear terminal deoxynucleotidyl transferase, TdT) have been used to identify leukaemic blasts (McCaffrey *et al.*, 1975; Bollum, 1979; Janossy *et al.*, 1980). In this study indirect immunofluorescence (IF) and immunoperoxidase (peroxidase-anti-peroxidase; IP-PAP) techniques were used to determine the optimal method for identifying $TdT^+$ leukaemic blasts in testicular tissue.

### MATERIALS AND METHODS

*Preparation of tissue biopsies*

A total of 40 testicular biopsies were examined from 25 patients receiving maintenance therapy for ALL. Some of these samples had been processed in other laboratories for conventional histology. Samples of normal thymus obtained from children undergoing cardiac surgery were used for positive controls. Negative control tissue included tonsils, collected after elective surgical removal, and post-mortem samples of normal testes from non-leukaemic individuals. The tissues were prepared in the following manner:

(*i*) *Touch preparations*.—The cut surface of biopsies was dabbed on to clean glass slides and allowed to dry at room temperature (RT) and fixed before staining in cold methanol at 4°C for 30 min or in cold formol-sucrose for 2 h.

(*ii*) *Cytocentrifuge preparations* (*cytospin*).—Marrow and blood leucocytes were separated on Ficoll-Triosil, resuspended and washed in phosphate buffered saline (PBS). pH 7·2. Smears were made in a cytocentrifuge and dried rapidly at RT. These cytospin preparations were fixed in cold methanol at 4°C (30 min) unless otherwise stated. Thymocytes were obtained by teasing out finely cut infant thymus tissue; these were processed in a similar manner.

(*iii*) *Tissue fixation and paraffin embedding*.—Portions of tonsil, testis and thymus were fixed in various solutions (Table I) and processed to paraffin wax. The most frequently used procedure was fixation in formol-sucrose (6 h, at 20°C) followed by incubation in successive washes of alcohol (3 × 2 h) and chloroform (2 × 2 h) prior to vacuum and wax embedding. It was possible to shorten each incubation to 1 h. Sections were washed in PBS (20 min) before testing in the IF or IP-PAP staining systems.

(*iv*) *Frozen material*.—Unfixed samples of thymus and leukaemic infiltrated testis were embedded in OCT compound (Miles Laboratories) and snap-frozen in isopentane (2-methyl butane) and liquid $N_2$. These were stored at −70°C or in liquid $N_2$.

*Enzyme digestion*

0·1% trypsin (BDH) in 0·1% calcium chloride (pH 8·6) and 0·1% deoxyribonuclease (DNase) in 0·1M sodium acetate buffer with 0·005M $MgCl_2$ (pH 7·4) were standardized for optimum digestion of cell and nuclear protein. The degree of trypsin or DNase digestion was assessed with methyl-green pyronin and Feulgen staining respectively (see below). Both enzyme solutions were freshly prepared and used immediately; the magnesium and calcium buffers were stored at 4°C.

*Antisera*

All antisera were used in sufficient amounts to cover the cell preparations on slides (10 μl on cytocentrifuge smears and 30–40 μl on sections). The incubation was carried out in moist chambers in order to prevent the evaporation of reagents. Rabbit anti-calf TdT (R-anti-TdT) antibody was purified on a TdT immunoadsorbent column (Bollum, 1975). This antibody was used at 0·1 μg/μl. A similar reagent is available from Bethesda Research Laboratories, Bethesda, Maryland. The reactivity pattern of this reagent has been extensively characterized in normal and leukaemic tissues. Nuclear TdT is expressed by 65–70% of normal infant thymocytes and 0·5–6%, non-B, non-T cells in normal or regenerating marrow. It is absent from normal peripheral lymphoid tissue and other organs. TdT can be demonstrated in blast cells of the common form of non-T, non-B ALL (Janossy *et al.*, 1980) thymic ALL (Bradstock *et al.*, 1980, 1981) and lymphoid blast crisis of chronic granulocytic leukaemia (Janossy *et al.*, 1979). It is not present in other forms of myeloid, T- or B-cell leukaemias (Janossy *et al.*, 1980).

In the IF test, a purified goat anti-rabbit IgG ($Fab_2$) antibody coupled to fluorescein isothiocyanate (G-anti-RIg-FITC; 1μg/μl) was used (Janossy *et al.*, 1979). In the IP-PAP test (Burns *et al.*, 1974), swine anti-rabbit IgG (Sw-anti-RIgG: Dako) second-layer was used at a 1:50 dilution, followed by rabbit peroxidase anti-peroxidase (PAP; Miles-Yeda) at 1:50 dilution.

*Staining procedure*

IP-PAP and IF staining were performed on pairs of samples. Staining was carried out at RT or at 4°C. In the IP-PAP test, endogeneous peroxidase was blocked with 0·3% $H_2O_2$ (w/v 30%) in methanol for 30 min and washed in PBS (10 min). Sections were treated with normal swine serum (NSS) 1:30 for 30 min, to reduce non-specific staining. Without further washing R-anti-TdT antibody was applied for 4 h at RT or overnight at 4°C. After 20–30 min wash in PBS, Sw-anti-RIgG second-layer antibody was added for 30 min at RT. After a further wash in PBS (30 min), the samples were stained for 30 min with rabbit PAP conjugates. The peroxidase activity was developed by incubating the slides in 1 mg/ml diaminobenzidine (DAB, Sigma) for 2 min after which 1 drop of $H_2O_2$ was added and incubated for a further 3 min. Sections were washed in tap water, dehydrated in alcohol, cleared in xylene and mounted. Some sections were counterstained with light green (0·05%, 30 sec) and examined under a light microscope. Other sections were studied without counterstaining under phase contrast. Parallel samples were processed by the indirect IF technique immediately after enzyme digestion. After incubation with R-anti-TdT, the preparations were washed for 30 min, incubated with G-anti-R-IgG-FITC and re-washed for 30 min. The glycerol-mounted samples were examined under a Zeiss fluorescence microscope.

To process the frozen tissue samples, 6μm cryostat sections were mounted on to glass microscope slides and immediately fixed for 15 min in pre-cooled (~0°C) 10% buffered formalin *within* the cryostat. Sections were washed in PBS (30 min) at RT and treated for IF and IP staining as described above. Neither method required DNase enzyme digestion.

To evaluate the staining specificity of the test layer, R-anti-TdT antibody was replaced by NSS (previously absorbed with human tonsil) at 1:50 dilution. Additional controls in the IP-PAP test involved omission of the second antibody, as well as sections stained with DAB only, with and without $H_2O_2$. The latter was to test the efficacy of blocking endogenous peroxidase.

### RESULTS

### *Effects of fixation on TdT staining of isolated thymocytes*

Cytospin preparations of thymocyte suspensions were fixed in various fixatives for different times (Table I). Excellent labelling of nuclear TdT was obtained when the smears were fixed in 6% formol-sucrose for up to 4 h at RT and up to 16 h at 4°C. Similarly, good staining was seen after fixing in 10% formalin for up to 2 h at RT or up to 4 h at 4°C. The intensity of staining was comparable to the strong labelling seen on the smears fixed in cold methanol by the conventional method. In contrast, Bouin's and Carnoy's fixatives quickly destroyed the antigenicity of TdT.

### *Effects of enzyme treatment on TdT staining in paraffin-embedded tissue sections*

The experiments above suggested that 10% formalin and 6% formol-sucrose may

TABLE I.—*Effects of fixation on the demonstration of TdT in cytocentrifuge preparations of human thymocytes*

| | 6% Formol-sucrose | | 10% Buffered formalin | | Bouin's | | Carnoy's | |
|---|---|---|---|---|---|---|---|---|
| Fixative | 20°C | 4°C | 20°C | 4°C | 20°C | 4°C | 20°C | 4°C |
| 30 min | N.T. | N.T. | N.T. | N.T. | − | ± | − | − |
| 1 h | + | + | + | + | − | − | − | − |
| 2 h | + | + | ++ | + | − | − | − | − |
| 3 h | ++ | + | ± | ++ | − | − | − | − |
| 4 h | + | ++ | ± | + | − | − | − | − |
| 16 h | + | + | − | ± | − | − | − | − |

*Staining*

++ good (see Fig. 1)
+ positive
± weak
− none
N.T. Not tested.

FIG. 1.—Comparative analysis of staining for TdT in DNase treated sections obtained from formalin-fixed, paraffin-embedded human thymus sample (a) and frozen thymus sample (b) or from frozen testis with leukaemic infiltrate (c). Sections (b) and (c) were fixed in formalin within the cryostat (see Methods) but were not treated with DNase. Cortical areas [c] contain abundant $TdT^+$ cells (nuclear staining). In the thymic medulla [m] few cells (arrows) are $TdT^+$ and most lymphoid cells are $TdT^-$. Note that tissue preservation is slightly better in "a" but is still acceptable in (b) and (c). The TdT staining is nevertheless more intense in (b) and (c) than in (a).

be suitable fixatives. Small (5 × 5mm) blocks of infant thymus were therefore placed in these fixatives for 4 h and processed to paraffin wax. Cut sections were then incubated overnight with R-anti-TdT. In both samples stained with the IP-PAP method, a clear but very weak nuclear staining was seen in the thymic cortex. In some parts of the samples fixed in 6% formol-sucrose, the staining appeared to be cytoplasmic.

In the following experiments, samples fixed in 6% formol-sucrose or 10% formalin (6 h) were treated with 0·1% DNase (Sigma, London, U.K.: Batch DN100. Lot 129C-0070) for 30 min at RT. After incubation, the Feulgen staining revealed only pale magenta nuclear remnants. Accordingly, the IP staining for TdT in these samples was much stronger than in the untreated samples (see above). A clear brown precipitation product was visible in the nuclei of cortical cells, whilst the majority of medullary thymocytes remained negative. The 5–10% $TdT^+$ cells in the medulla appeared to have genuine nuclear positivity, and could correspond to recent arrivals from the cortex (Fig. 1). Longer incubations with DNase, not only abolished nuclear Feulgen positivity but also removed the nucleus from the cell. Thus the titration of DNase was critical for obtaining optimal results. Trypsinization of sections for 15–30 min also increased the IP staining of nuclear TdT, though less effectively than optimal

DNase treatment. Longer incubation with trypsin (1–2 h) abolished the TdT staining and damaged tissue morphology.

*Investigation of leukaemic infiltrates in testis*

These formalin-fixed and paraffin-embedded samples were used with the optimal DNase digestion. The sections were stained

TABLE II.—*Histological and immunohistological (TdT) analysis of paraffin-embedded testicular biopsies taken from 25 cases of ALL in remission*

| Cases | Histology (presence of leukaemic infiltrate) | Staining for TdT IF or IP* |
|---|---|---|
| 1–8 | + | + |
| 9 | + | (+) |
| 10 | + | − |
| 11–12 | ? | − |
| 13–17 | − | (+) |
| 18–19 | − | + |
| 20–25 | − | − |

* Immunofluorescence of immunoperoxidase. Cases 6–8 contained blasts heterogeneous in respect of TdT staining (Fig. 3).
(+) Minimal leukaemic infiltrate.

for TdT with both the IP-PAP and IF methods (Table II). In 10 patients (cases 1–10) the routine histology of testicular biopsies showed heavy leukaemic infiltrates. In 9 of these, unequivocal nuclear TdT staining was visible in the infiltrating lymphoblasts (Figs 2 & 3) while in one case no TdT$^+$ blasts were seen. In 3 of the 8 immunologically positive cases, some of the leukaemic blasts were TdT$^-$. In these tissues, the lymphoblasts showed clear heterogeneity in their staining patterns; some contained no identifiable TdT, though near to strongly TdT$^+$ blasts (Fig. 3).

In all heavily infiltrated samples, the preservation of morphological details was remarkable. The TdT positivity corresponded to the *nucleus* and clear cytoplasmic staining was seen only in the dividing cells, where TdT is known to be released from the disrupted nucleus (Fig. 3). Some TdT$^+$ cells had cigar-shaped nuclei and represented elongated migrating forms (Fig. 2) which were easily overlooked in the conventional histological prepara-

FIG. 2.—Analysis of paraffin-embedded testicular biopsies with immunoperoxidase. (a) Negative-control preparations were incubated with second layers only but not with anti-TdT serum. The lymphoblasts (arrows) are peroxidase negative. (b) Adjacent section to (a) staining for TdT. Infiltrating blasts are heavily labelled. (c) In this sample, the scattered TdT$^+$ blasts could not be identified with conventional histology. St: Seminiferous tubules.

FIG. 3.—Analysis of TdT with immunofluorescence. The same paraffin-embedded sample of involved testis was photographed with phase (A) and fluorescein filter (FITC; B). Some blasts have characteristically shaped nuclei, clearly stained $TdT^{+}$. Note that the nucleoli remain unstained ("holes" in TdT stain). The blasts are clearly heterogeneous; some being $TdT^{-}$ (small arrows). Dividing cells release the TdT into the cytoplasm (large arrow). (C) Cellular prints obtained by "dabbing" the freshly cut surface of infant thymus on to glass slides. (D) Cytospin preparation of leukaemic lymphoblasts. Both C and D were stained for TdT, and show comparable morphology to B. Small arrows point to $TdT^{-}$ thymocytes and weakly $TdT^{+}$ positive blasts. Large arrow denotes a dividing blast.

tions. Other cells contained small $TdT^{-}$ "holes" *within* the nucleus which corresponded to the nucleolus. These patterns in some blasts have the appearance of a pseudo-lobulated nucleus (Fig. 2).

The details of TdT staining of leukaemic blasts in formalin-fixed testicular biopsies were comparable to the excellent staining obtained in cytocentrifuge preparations of blood-borne leukaemic blasts. Similarly, the morphological details were comparable to the quality of thymocyte preparations obtained by impressions of freshly cut thymic tissue on glass slides (Fig. 3).

*Frozen tissue sections*

Using cryostat sections of thymus mounted and fixed at 0°C, clear nuclear TdT localization was obtained by both IP-PAP and IF methods. The intensity of TdT staining was stronger in the frozen tissue than in the DNase-digested paraffin-embedded sections (Fig. 1b). Moreover, the test on frozen material required smaller amounts of anti-TdT reagent. Similarly, in the frozen testicular material with overt leukaemic infiltration, intense $TdT^+$ blasts were seen throughout the tissue (Fig. 1c). It must be emphasised, however, that TdT staining is inferior in "conventionally" prepared cryostat sections mounted on glass slides and fixed at RT. This is because TdT, a soluble protein, appears to "diffuse out" of the nucleus, causing smudged cytoplasmic staining. Thus the rapid fixation after cutting is important.

*Investigation of minimal leukaemic infiltrates in testes*

Testicular biopsies from 7 patients (cases 13–19) showed the normal structure of seminiferous tubules, with *no histological evidence* of increased lymphoid or lymphoblastic infiltration (Table II). Using immunological markers, however, scanty but widespread $TdT^+$ cells were identified in the interstitial tissue of 5 cases (13–17). In Case 18, a few well defined $TdT^+$ cells were seen near the capsule. Similarly, in Case 19, a group of $TdT^+$ cells was seen in the subcapsular area (Fig. 2). When these cells were examined with a $\times 100$ oil objective, characteristic patchy IF staining and IP complexes could be seen in the "crevasses" between the ridges of nuclear chromatin bands. This pattern is typical of TdT staining. It is most noticeable in thymocytes where the amount of detectable TdT is moderate or low. A somewhat finer meshwork is seen in individual marrow $TdT^+$ cells and leukaemic blasts, which contain higher amounts of TdT (Figs 2 & 3). It is important to point out that in touch preparations of these minimally infiltrated biopsies, we could *not* identify the infiltrating $TdT^+$ cell population.

No identifiable $TdT^+$ cells were present in the testicular biopsies from another 6 patients (cases 20–25) which showed no histological evidence of leukaemic infiltration.

*Negative controls*

No $TdT^+$ cells were seen in the negative-control samples of thymus and post-mortem testicular tissue. An additional negative control for IP-PAP and IF techniques was applied to both test and control preparations, using second-layer antibodies but without the R-anti-TdT reagent. This was performed on sections adjacent to those used for positive staining with R-anti-TdT. No $TdT^+$ cells were labelled by the second-layer antibodies alone (Fig.2).

*Comparison of IP-PAP analysis in paraffin-embedded tissue*

Using optimal methods, both peroxidase and fluorescence analysis gave reliable results. The advantages of the IP-PAP technique were 2-fold. The permanent preparations allowed careful analysis of nuclear details with high magnification on $TdT^+$ cells (Figs 1 & 3). Counterstaining with methyl green, though not essential with phase-contrast microscopes, helped the comparison with conventional H & E staining. The IP-PAP method is nevertheless more difficult to standardize, and takes longer to perform, than IF staining.

IF staining was quicker and easier but had two disadvantages. Autofluorescent red cells had to be distinguished from nuclear TdT staining. This was accomplished by demonstrating red-cell fluorescence on *both* the fluorescein (green) and rhodamine (red) fluorescence channels. This contrasted with the genuine TdT staining, which was visible only on the fluorescein (FITC, green) channel. A more serious drawback was the fading of IF

staining, with a lack of permanency of the preparations.

### DISCUSSION

In normal human tissues $TdT^+$ cells can only be found in marrow and thymus. At any other site, $TdT^+$ cells suggest residual ALL. This finding has recently been exploited to detect ALL cells in the cerebrospinal fluid (Bradstock *et al.*, 1980). In this study we have standardized the method to investigate residual testicular ALL.

Three factors have proved to be important in analysing paraffin-embedded material. First, only batches of highly purified specific anti-TdT antibody eluted from TdT immunoadsorbant columns gave sufficiently clear results. Second, formalin or formol-sucrose was superior to other fixatives such as Bouin's or Carnoy's for biopsy material, and produced clear nuclear TdT localization. Third, it was essential to digest the sections of paraffin-embedded material with DNase. In contrast, however, sections of *frozen* biopsies could be stained very strongly with smaller amounts of anti-TdT serum, without DNase digestion. Thus, it is advisable to prepare frozen samples of all biopsies. Finally, the analysis of tissue sections was more sensitive for detecting rare blasts than the touch preparations from the same samples.

One advantage of this extension of the TdT method is that it may be feasible to perform retrospective studies on samples fixed and embedded by routine histological methods. The staining for TdT can clearly detect scanty ALL blasts *scattered* in the interstitial testicular tissue (Fig. 3) and not forming overt cell clusters. It is therefore possible that TdT staining is a more sensitive method than conventional histology in this specialised tissue. The storage of frozen samples is also important in laboratories aiming to perform TdT staining, because this will simplify (and thus increase the reliability of) the procedure.

The significance of TdT analysis in testicular biopsies is as follows. In one series of 170 biopsies performed during or at the end of maintenance ALL therapy, 18 samples were frankly positive for leukaemic cells by routine histology (Eden & Rankin, unpub.). The observations on a further 17 samples (10%) were difficult to interpret because of the presence of inflammatory and large mesenchymal cells or "crush" artefacts. In addition, 13 samples (7%) were reported as histologically negative, but subsequently relapsed in the testis. The median time between these biopsies and the relapses was 12 months. These "false negative" samples might be due to a missed focal deposit. We have already observed one such case, where $TdT^+$ cells were detected in the subcapsular area (Table II) and the patient relapsed 4 months later. Alternatively, relapse may derive from a diffuse cellular infiltrate which, when scanty, might be difficult to notice without a specific marker. In this respect, it is important to point out that even where a definite infiltration is diagnosed histologically, it is most frequently diffusely distributed and not focal (Eden & Rankin, unpub.; Kuo *et al.*, 1976; Lenden *et al.*, 1978). In conclusion, this paper demonstrates that the immunohistological analysis of $TdT^+$ cells has an important role in identifying minimal testicular leukaemia of non-T, non-B and T-ALL types.

Supported by Grants RJ8A from Research Fund (to technical assistant), Grant G. 978/443 from the Medical Research Council of Great Britain (to Dr J. A. Thomas) and Grant CA 23262 from the National Cancer Institute (to Dr F. J. Bollum).

We thank Mrs Wendy Lake for her technical assistance.

### REFERENCES

Bollum, F. J. (1975) Antibody to terminal deoxynucleotidyl transferase. *Proc. Natl. Acad. Sci.*, **72**, 4119.

Bollum, F. J. (1979) Terminal deoxynucleotidyl-transferase as a hemopoietic cell marker. *Blood*, **54**, 1203.

Bradstock, K. F., Janossy, G., Pizzolo, G. & 6 others. (1980) Subpopulations of normal and leukemic human thymocytes: An analysis with the use of monoclonal antibodies. *J. Natl Cancer Inst.*, **65**, 33.

BRADSTOCK, K. F., PAPAGEORGIOU, E. S. & JANOSSY, G. (1981) Diagnosis of meningeal involvement in patients with acute lymphoblastic leukemia using immunofluorescence for terminal transferase. *Cancer*, **47**, 2478.

BURNS, J., HAMBRIDGE, M. & TAYLOR, C. R. (1974) Intracellular immunoglobulins: A comparative study in three standard tissue processing methods using horseradish peroxidase and fluorochrome conjugates. *J. Clin. Pathol.*, **27**, 548.

EDEN, O. B., HARDISTY, R. M., INNES, E. M., KAY, H. E. M. & PETO, J. (1978) Testicular disease in acute lymphoblastic leukemia in childhood. *Br. Med. J.*, **i**, 334.

JANOSSY, G., WOODRUFF, R. K., PIPPARD, M. J. & 5 others. (1979) Relation of "lymphoid" phenotype and response to chemotherapy incorporating vincristine-prednisolone in the acute phase of $Ph^1$ positive leukemia. *Cancer*, **43**, 426.

JANOSSY, G., BOLLUM, F. J., BRADSTOCK, K. F. & ASHLEY, J. (1980) Cellular phenotypes of normal and leukemic haemopoietic cells determined by selected antibody combination. *Blood*, **56**, 430.

KIM, T. H., LIU, V-KS., WOODRUFF, R. D. & RAGAB, A. H. (1979) Testicular biopsy prior to termination of leukemic therapy. *J. Paediatr.*, **94**, 95.

KUO, T., TSCHAN, T-P. & CHU, J-Y. (1976). Testicular relapse in childhood during bone marrow remission. *Cancer*, **38**, 2604.

LENDEN, M., HAN, I. M., PALMER, M. K., SHALET, S. M. & MORRIS-JONES, P. H. (1978) Testicular histology after combination chemotherapy in childhood for acute lymphoblastic leukaemia. *Lancet*, **ii**, 439.

McCAFFREY, R. P., HARRISON, A., PARKMAN, B. S. & BALTIMORE, D. (1975) Terminal deoxynucleotidyl transferase activity in human leukemic cells and normal thymocytes. *N. Engl. J. Med.*, **292**, 775.

MILLER, D. R. (1980) Acute lymphoblastic leukemia. *Ped. Clin. North. Am.*, **27**, 269.

PINKEL, D. (1976) Treatment of acute leukemia. *Ped. Clin. North. Am.*, **23**, 117.